# Inflammation severity, rather than respiratory failure, is strongly associated with mortality of ARDS patients in high-altitude ICUs

**DOI:** 10.3389/fphys.2024.1520650

**Published:** 2025-01-15

**Authors:** Daniel Molano-Franco, Joan Ramon Masclans Enviz, Antonio Viruez-Soto, Mario Gomez, Harvey Rojas, Edgar Beltran, Victor Nieto, Fernanda Aliaga-Raduan, Pablo Iturri, Christian Arias-Reyes, Jorge Soliz

**Affiliations:** ^1^ High Altitude Intensive Care Medicine International Group (GIMIA), La Paz, Bolivia; ^2^ High Altitude Intensive Care Medicine International Group (GIMIA), Lima, Peru; ^3^ High Altitude Intensive Care Medicine International Group (GIMIA), Bogotá, Colombia; ^4^ Critical Care Department, Hospital San Jose, Foundation University of Sciencies Health- CIMCA Research Group, Bogota, Colombia; ^5^ Critical Care Department, Center of Treatment and Investigation of Cancer- CTIC- GRIBOS Research Group, Bogota, Colombia; ^6^ Critical Care Department, Hospital del Mar Research Institute – (IMIM), Universitat Pompeu Fabra (UPF), Barcelona, Spain; ^7^ Department of Medicine and Life Sciencies (MELIS), Universitat Pompeu Fabra (UPF), Barcelona, Spain; ^8^ Critical Care Department, Hospital Agramont, El Alto, La Paz, Bolivia; ^9^ Centre de Recherche de l’Institute Universitaire de Cardiologie et de Pneumologie de Québec, Université Laval, Québec City, QC, Canada; ^10^ Bolivian Foundation of Altitude Sciences (BFAS), Brain Research Institute, La Paz, Bolivia; ^11^ Center for Integrative Brain Research, Seattle Children’s Research Institute, Seattle, WA, United States

**Keywords:** ARDS, high-altitude, mortality, inflammatory markers, hypobaric hypoxia, high altitude adaptation, ICU

## Abstract

**Introduction:**

In high-altitude cities located above 2,500 m, hospitals face a concerning mortality rate of over 50% among intensive care unit (ICU) patients with acute respiratory distress syndrome (ARDS). This elevated mortality rate is largely due to the absence of altitude-specific medical protocols that consider the unique physiological adaptations of high-altitude residents to hypoxic conditions. This study addresses this critical gap by analyzing demographic, clinical, sex-specific, and preclinical data from ICUs in Bogotá, Colombia (2,650 m) and El Alto, Bolivia (4,150 m).

**Methods:**

A cohort of seventy ARDS patients, aged 18 and older, was evaluated within 24 h of ICU admission. Data collected included demographic information (age, sex), clinical characteristics (primary pathology, weight, height), vital signs, respiratory variables, cardiorespiratory parameters, blood count results, inflammatory markers, severity assessment scores, and comorbidities. Advanced statistical analyses, such as multivariate logistic regression and principal component analysis, were utilized to identify key clinical predictors of ARDS-related mortality.

**Results:**

Our findings indicate that in high-altitude ICUs, monitoring inflammatory markers may be more beneficial for improving ARDS survival rates than emphasizing respiratory failure markers. Unexpectedly, we found no significant differences in clinical outcomes between altitudes of 2,650 and 4,150 m or between male and female patients.

**Conclusion:**

The study concludes that, in high-altitude settings, ARDS patient survival in ICUs is more closely associated with managing inflammatory responses than with focusing solely on respiratory parameters. Further large-scale studies are recommended to validate the impact of inflammatory marker monitoring on survival outcomes in high-altitude ICUs.

## Introduction

The syndromic definition of Acute Respiratory Distress Syndrome (ARDS) encapsulates a spectrum of pulmonary physiological abnormalities and chest radiographic findings. Furthermore, it involves variations in biological pathways of injury, supported by evidence from plasma protein biomarkers and gene expression profiles (Force et al., 2012). In the low-altitude Intensive Care Unit (ICU) settings (ranging from 0 to 1,500 m), the clinical outcomes of ARDS patients requiring invasive mechanical ventilation primarily rely on the effectiveness of ventilatory support (Rubenfeld et al., 2005; Bellani et al., 2016). Inadequate respiratory variables, associated to partial arterial oxygen pressure (PaO_2_) to fraction of inspired oxygen (Fio2) ratio often correlates with elevated mortality rates in such cases (Rubenfeld et al., 2005; Bellani et al., 2016). Various indicators predictive of progression towards a fatal outcome include parameters such as the Partial Pressure of Arterial Oxygen to Fraction of Inspired Oxygen ratio (PAFI), the Saturation of Arterial Oxygen to Fraction of Inspired Oxygen ratio (SAFI), as well as scores derived from the Sequential Organ Failure Assessment (SOFA) and Acute Physiology and Chronic Health Evaluation (APACHE) systems, all of which serve as respiratory indices. Additionally, markers of inflammation, including cytokine levels and C-reactive protein (CRP) levels, are integral in discerning the clinical trajectory of ARDS patients (Calfee et al., 2015; Ortiz et al., 2022).

At elevations exceeding 2,500 m (high altitude), a reduction in barometric pressure (BP) results in approximately one-third reduction in PaO_2_. Despite this, more than 150 million people worldwide reside above high altitude (Arias-Reyes et al., 2021a; Zubieta-DeUrioste et al., 2023). Furthermore, prominent urban centers in Latin America, like La Paz (Bolivia), Quito (Ecuador), Bogotá (Colombia), and Mexico City (Ciudad de México, Mexico), are situated at high altitudes and collectively house more than 38,181,350 people (Zubieta-DeUrioste et al., 2023).

To counteract the reduced PaO_2_ levels to which permanent high-altitude residents are exposed, physiological, cellular, and molecular adaptations occur to maintain the balance between oxygen supply and demand (Arias-Reyes et al., 2021a). These adjustments primarily involve increased ventilation (regulated by the respiratory control system and carotid bodies), erythropoiesis-mediated rise in red blood cell count (Soliz et al., 2005; Zubieta-Calleja et al., 2007), generalized vasodilation, and angiogenesis (regulated by vascular endothelium angiogenetic factors) (Scheinfeldt and Tishkoff, 2010). These significant physiological differences between residents at sea-level and those at high-altitudes raise concerns about the appropriateness and safety of applying guidelines that do not consider high-altitude ICU settings. In fact, given that PaO_2_ levels of 50–67 mmHg and peripheral oxygen saturation (SatpO_2_) of 89%–95% are considered normoxemic values in permanent high-altitude residents (Viruez-Soto et al., 2022; Viruez-Soto et al., 2020), current high-altitude medical guidelines lack specific markers conducive to reducing the high mortality rate, approximately 50% of ARDS patients in the ICU (Arias Ramos et al., 2022). Hence, in this study, we analyzed respiratory, hematological, inflammatory, and comorbidity clinical data of ICU patients diagnosed with ARDS from Bogotá, Colombia, and El Alto, Bolivia, located 2,640 and 4,150 m above sea level, respectively. The aim was to identify clinical markers that could facilitate a more effective therapeutic approach and improve survival outcomes. Our findings strongly suggest that in high-altitude settings, ICU survival among ARDS patients primarily depends on monitoring inflammatory parameters rather than respiratory failure. By prioritizing monitoring of inflammatory markers, there is potential to significantly improve survival rates in these settings. These results emphasize the necessity of redefining high-altitude pathophysiology, especially in ICUs, where a comprehensive understanding is vital for patient survival.

## Methods

An ambispective cohort study was carried out in patients admitted to the ICU with a diagnosis of ARDS in two third-level hospitals located in Colombia and Bolivia: University Hospital San José de Bogotá (Altitude: 2,640 m above sea level) and University Hospital Agramont from El Alto city in Bolivia (Altitude: 4,150 m above sea level). Data collection spanned retrospectively from April 2022 to April 2023 and prospectively from April to June 2023, extracted from electronic medical records covering the hospitalization period. The study was conducted using data collected from the initial day of ICU admission until the patient’s death or discharge from the ICU, with a maximum follow-up period of 28 days after admission. Obtaining post-discharge records was not considered due to difficulty in maintaining contact with discharged patients. The protocol for this study, numbered 691–2023, was approved by the Ethical Committee of Hospital San Jose and the Hospital Agramont.

### Patients

Seventy patients aged 18 years or older, who were within at least 24 h of ICU admission and had a confirmed diagnosis of ARDS due to pulmonary sepsis (bacterial or viral), extrapulmonary sepsis, or other causes such as acute pancreatitis, trauma, massive transfusion, or food aspiration, were enrolled. The diagnosis of ARDS was aligned with the Berlin definition criteria of acute hypoxemic respiratory failure, onset within 7 days of the insult or new or worsening respiratory symptoms, bilateral opacities on chest imaging that are not completely explained by other factors, and cardiac failure not the primary cause of acute respiratory failure (Ferguson et al., 2012). During the first 24 h of admission to the ICU, tidal volume, plateau pressure, positive end-expiratory pressure (PEEP), peak pressure and driving pressure were evaluated. Patients receiving invasive mechanical ventilation with simultaneous measurements of arterial blood gases and peripheral oxygen saturation (SpO_2_) were included, regardless of saturation levels, disease severity, ventilatory mode, vasopressor support, or radiological findings. Exclusions involved patients with concurrent myelodysplastic syndrome or anemia, terminal illness, extracorporeal membrane oxygenation, and those lacking reported data on FiO_2_, PaO_2_, and SpO_2_ in clinical records.

### Variables

Collected data included age, sex, primary pathology, weight, height, vital signs, hemoglobin concentration, blood erythropoietin concentration, hematocrit, C-reactive protein (CRP) concentration, lactate concentration, white blood cell count, sequential organ failure assessment (SOFA) score, acute physiology, general health condition, assessment severity score (APACHE II), vasopressor and ventilatory support parameters, arterial blood gas values, oxygenation indices, duration of mechanical ventilation, ICU, and hospital stay. To ensure accuracy, two members of the research team abstracted the data and reviewed it twice. Approval was obtained from the ethics committee of Hospital San José and Hospital Agramont de El Alto.

### Statistical analysis

An initial descriptive analysis summarized the categorical and respiratory variables. The former were presented as frequencies and percentages, while the continuous data were presented as means and interquartile ranges. Differences between survivors and deceased patients were initially explored by univariate statistical analyses. Qualitative variables were compared using chi-square tests and quantitative variables using the Student’s t-tests adjusted for non-homogeneous variance when needed. These analyses were performed in IBM SPSS Statistics for Windows, Version 26.0. Armonk, NY: IBM Corp.

Principal component analysis (PCA) was performed using the data of 35 quantitative continuous variables: age, altitude, SOFA score, APACHE II score, weight, body mass index (BMI), mean arterial pressure (MAP), heart rate, breathing frequency, temperature, FiO_2_, PaO_2_, PaCO_2_, SaO_2_, PaO2/FiO2, SaO2/FiO2, total leukocyte count, total erythrocyte count, total lymphocyte count, total neutrophil count, hemoglobin concentration, hematocrit, mean corpuscular volume (MCV), mean corpuscular hemoglobin (MCH), red cell distribution width (RDW), total platelet count, % neutrophils, % basophils, % lymphocytes, % monocytes, % eosinophil, erythropoietin concentration (EPO), lactate concentration, C-reactive protein concentration (CRP), and days spent in the ICU. The principal components with eigenvalues greater than 1.0 were selected following the Kaiser rule (Ben Salem and Ben Abdelaziz, 2021).

A multivariate logistic regression of the first 10 principal components (PC) was performed to evaluate the effect of each of these PCs on the probability of death using the model: Probability of death ∼ Intercept + PC1 + PC2 + PC3 + PC4 + PC5 + PC6 + PC7 +PC8 + PC9 + PC10. The classification cutoff was set to 0.5.

Simple logistic regressions were used to assess the effect of the clinical variables identified as relevant from the PCs above on mortality. Sixteen variables tested were the following: age, SOFA score, APACHE II score, weight, BMI, total leukocyte count, total lymphocyte count, total neutrophil count, hemoglobin concentration, red cell distribution width (RDW), % neutrophils, % basophils, % lymphocytes, % monocytes, EPO, and CRP. For each analysis, the classification cutoff was set to 0.5.

PCA and logistic regression analyses were performed using GraphPad Prism version 10.0.0 for Windows, GraphPad Software, Boston, Massachusetts, United States.

## Results

### Demographic description of the sample

A cohort of 70 patients diagnosed with ARDS, according to the Berlin consensus criteria implemented by institutional protocols or hospitals, were included in this study upon admission to the ICU. The cohort consisted of 34 women and 36 men. Among them, 52 patients were recruited from San José Hospital in Bogotá (Altitude: 2,640 m), while 18 patients were from Agramont Hospital in El Alto, Bolivia (Altitude: 4,150 m). The study revealed an overall mortality rate of 40% within the cohort. Our analysis did not identify any correlation between demographic factors and mortality rates. (Table 1).

**TABLE 1:** Demographic factors.

	Deceased (n = 28)		Survivors (n = 42)		
	Mean	IQR	Mean	IQR	t-test *p*-value
Age (yrs.)	62.2	56.0–75.0	60.5	53.3–71.3	0.6
Weight (kg)	61.3	50.5–65.8	66.2	58.8–76.0	0.9
BMI (kg/m^2^)	23.9	20.0–25.6	24.7	22.2–27.1	0.7
	%		%		χ^2^-test *p*-value
Sex					0.63
Female (n = 34)	21.4		27.1		
Male (n = 36)	18.6		32.9		
City of residence					0.22
Bogota (n = 52)	32.8		41.4		
El Alto (n = 18)	7.1		18.6		

### Respiratory parameters between deceased and surviving ARDS patients in the ICU did not demonstrate statistically significant differences

During the first 24 h of ICU admission—a critical period that often determines survival—key ventilatory parameters were assessed, including tidal volume, plateau pressure, positive end-expiratory pressure (PEEP), peak inspiratory pressure, and driving pressure. Notably, our analysis revealed no significant differences in these parameters between the two groups (Table 2), suggesting that early ventilatory settings may not be the primary determinants of mortality in this cohort.

**TABLE 2:** Respiratory variables.

Respiratory variable*	Survivors (42)	No survivors (28)	t-test p-value
Tidal Volume (mL)	480	480	0.91
Plateau Pressure (mmHg)	22	24	0.74
PEEP (mmHg)	8	10	0.23
Peak pressure (mmHg)	34	34	0.21
Driving pressure (mmHg)	12	10	0.25
*24 h to admission			

### Out of 48 clinical parameters analyzed, six exhibited significant differences between deceased and surviving ARDS ICU patients at high altitudes

**FIGURE 1:**
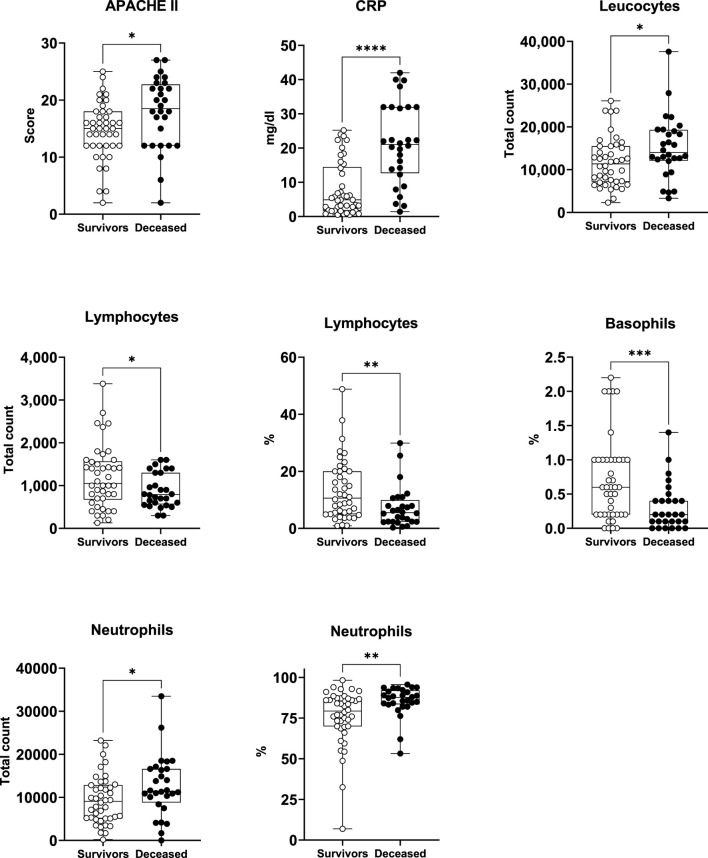
Survivors and deceased patients showed differences in 8 out of 59 studied variables. Seven of these parameters were markers of inflammation. *p < 0.05; **p < 0.01; ***p < 0.001; ****p < 0.0001. n_survivors_ = 42; n_deceased_ = 28.

CRP, leukocytes, lymphocytes, basophils, neutrophils, and APACHE II (Table 3; Figure 1). Specifically, the deceased individuals exhibited significantly elevated values of CRP, total leukocyte count, total count, and percentage of neutrophils, as well as APACHE II score (Figure 1). On the contrary, both the total count and the percentage of lymphocytes, along with the percentage of basophils, were significantly lower in deceased patients (Figure 1).

**TABLE 3:** Clinical parameters.

Cardiorespiratory function	Deceased (n = 28)		Survived (n = 42)		t-test
	Mean	IQR	Mean	IQR	p-value
MAP (mmHg)	78.8	68.0–88.0	80.2	71.5–86.3	0.72
Heart rate (BPM)	93.4	79.3–108.0	90.0	80.0–100.0	0.37
Breathing Frequency (BPM)	22.0	18.0–25.5	22.2	18.8–24.0	0.83
Temperature (°C)	36.8	36.3–37.3	36.7	36.2–36.8	0.46
FiO2 (mmHg)	71.6	50.0–100.0	60.9	36.0–85.0	0.09
pCO2 (mmHg)	57.8	53.5–61.5	59.5	54.8–62.0	0.30
pO2 (mmHg)	37.0	32.0–38.8	34.7	30.0–37.3	0.21
SaO2 (%)	89.3	88.0–92.0	90.4	89.0–92.0	0.28
PaFi (mmHg)	97.8	56.5–117.5	118.6	54.8–167.5	0.12
SaFi (mmHg)	144.1	88.0–179.3	172.4	105.5–248.5	0.10

Hemogram	Mean	IQR	Mean	IQR	p-value
Hematocrit (%)	36.5	28.2–42.1	38.7	29.5–45.6	0.41
Erythrocytes (mcL)	3,885.0	3,145.0–4,715.0	4,448.0	3,530.0–5,013.8	0.04
Hemoglobin (mg/dL)	11.6	9.0–13.6	12.6	9.6–15.3	0.21
RDW (%)	17.8	15.1–19.2	19.4	15.0–22.0	0.20
MCH (pg)	26.9	24.4–29.2	29.7	24.0–30.5	0.26
MCV (fL)	80.0	74.7–89.3	79.5	64.8–92.2	0.87
Leukocytes (mcL)	15,223.6	12,100.0–19,300.0	11,811.7	7,150.0–15,500.0	0.03
Lymphocytes (mcL)	886.0	562.5–1,300.0	1,197.0	675.0–1,566.3	0.04
Neutrophils (mcL)	12,613.5	8,825.0–16,600.0	9,392.5	5,100.0–12,883.0	0.04
Platelets (mcL)	258,500.0	133,250.0–336,250.0	313,214.3	204,750.0–380,250.0	0.18
% Neutrophils	85.6	83.5–92.1	75.9	69.9–87.1	0.01
% Basophils	0.3	0.1–0.4	0.7	0.2–1.0	0.00
% Lymphocytes	7.3	2.3–9.9	13.4	5.0–20.0	0.01
% Monocytes	4.1	1.2–5.5	6.2	2.0–8.0	0.14
% Eosinophils	0.5	0.0–0.9	0.9	0.0–1.0	0.25

	Deceased (n = 28)		Survived (n = 42)		t-test
Inflammation	Mean	IQR	Mean	IQR	p-value
C-reactive protein (mg/dL)	21.3	12.7–32.0	7.9	1.7–14.5	0.00
EPO (mUl/mL)	18.7	2.4–28.1	25.2	6.6–18.6	0.42

Metabolic acidosis	Mean	IQR	Mean	IQR	p-value
Lactate (mmol/L)	3.6	1.6–5.2	2.7	1.5–3.3	0.13

Scores	Mean	IQR	Mean	IQR	p-value
SOFA	6.8	4.0–8.8	5.4	3.0–8.0	0.07
APACHE II	17.9	12.0–22.8	14.6	12.0–18.0	0.20
Comorbidities	**%**		**%**		**χ2-test p-value**
Hypertension	53.6		45.2		0.49
Diabetes mellitus	14.3		26.2		0.23
Hyperlipidemia	25.0		21.4		0.73
Coronary disease	7.1		7.1		1.00
Cardiac failure	21.4		26.2		0.65
Asthma	3.6		4.8		0.81
COPD	39.3		40.5		0.92
Smoker	42.9		28.6		0.22
Cerebral vascular disease	10.7		4.8		0.34
Chronic kidney disease	17.9		11.9		0.49
Liver disease	7.1		100.0		0.08
Hypothyroidism	17.9		21.4		0.71
Immunosuppression	7.1		7.1		1.00
Cancer	3.6		2.4		0.77
Alcoholism	14.3		2.4		0.06
Special support					
Vasopressor support	89.3		45.2		<0.0001
Renal support	17.9		7.1		0.17

### Principal component analysis and multivariate logistic regression reveal that inflammatory rather than respiratory distress parameters are strongly associated with mortality in high-altitude patients

**FIGURE 2:**
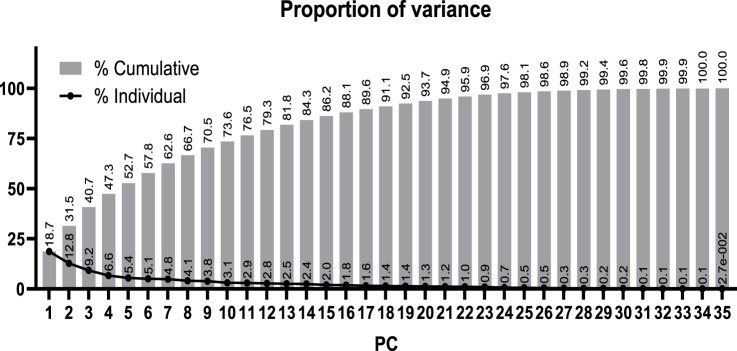
Proportion of the total variance from the clinical data explained by each calculated principal component (PC).

**FIGURE 3:**
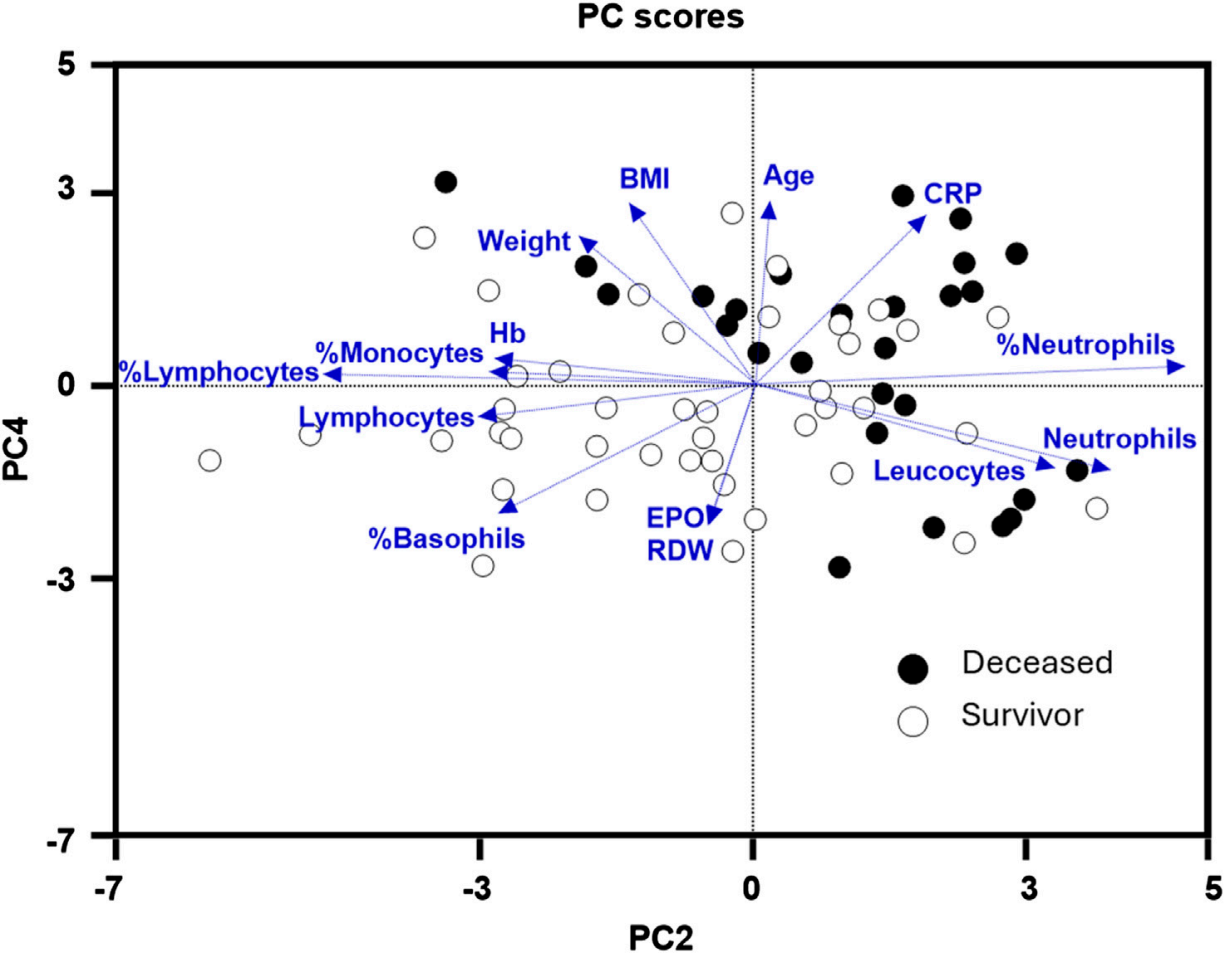
Principal components (PC) 2 and 4 were the best at identifying deceased (black dots) from survivor (white dots) high-altitude patients with subacute respiratory failure. Within these two PCs, hematological parameters: hemoglobin (Hb), erythropoietin (EPO), (RDW), and inflammatory markers: C-reactive protein, % monocytes, % lymphocytes, lymphocytes (total count), % basophils, leukocytes (total count), % neutrophils, neutrophils (total count), were the most relevant clinical variables in discriminating deceased from survivor patients.

Subsequently, our focus was on discerning whether specific combinations of clinical parameters could distinguish between patients who survived and those who did not. To achieve this, we performed Principal Component Analysis (PCA) using only the clinical variables that are measured as continuous numerical values. We analyzed a total of 35 variables and generated an equal number of principal components (Figure 2). Then, according to the Kaiser-Gutman rule (Norman, 1988), we prioritized PCs with eigenvalues equal to or greater than 1 and identified PCs 1 to 10 for further examination. These selected PCs were then utilized in a multivariate logistic regression, with survival as the dependent variable and the corresponding PC scores as independent variables. This analysis showed that only PC 2 and 4 exhibited significant predictive power for mortality (Table 4). Finally, through a second logistic regression, we used the clinical variables with contributions equal to or greater than 5% of PC 2 and 4 to identify the most effective clinical predictors of death (Tables 1, 3–6). Remarkably, our findings revealed that inflammatory parameters showed the strongest association with mortality, rather than indicators of respiratory oxygenation suggested by current medical guidelines based on sea level medical guidelines. Specifically, surviving patients, compared to deceased patients, showed higher levels of neutrophils (total count and percentage), leukocytes (total count), and C-reactive protein, along with older age and increased body weight. Furthermore, deceased patients showed reduced levels of lymphocytes (both total count and percentage), percentage of monocytes, percentage of basophils, hemoglobin concentration, EPO, and RDW (Figure 3).

**TABLE 4:** Predictive power of principal components (PC) for identifying surviving and deceased high-altitude patients with subacute respiratory failure admitted to ICU.

	*p*-value	OR	95% CI	AUC	95% CI
PC1	0.1	1.3	0.97–2.0	0.89	0.82–0.97
PC2	**0.001**	2.6	1.6–5.1	0.89	0.82–0.97
PC3	0.68	1.1	0.67–1.8	0.89	0.82–0.97
PC4	**0.01**	2.2	1.3–4.4	0.89	0.82–0.97
PC5	0.06	0.57	0.29–0.99	0.89	0.82–0.97
PC6	0.08	1.9	0.95–4.2	0.89	0.82–0.97
PC7	0.91	0.97	0.58–1.6	0.89	0.82–0.97
PC8	0.28	0.69	0.34–1.3	0.89	0.82–0.97
PC9	0.33	1.4	0.74–2.9	0.89	0.82–0.97
PC10	0.72	1.2	0.52–2.8	0.89	0.82–0.97

Only PCs, with eigen values equal or higher than 1 were used.

Significant p-values are shown in bold.

**TABLE 5:** Contribution of the studied variables to the variance explained by each principal component (PC).

PC1		PC2		PC3		PC4		PC5	
Eigenvalue = 6.5		Eigenvalue = 4.5		Eigenvalue = 3.2		Eigenvalue = 2.3		Eigenvalue = 1.9	
Variable	%	Variable	%	Variable	%	Variable	%	Variable	%
FiO_2_ (mmHg)	12.9	% Lymphocytes	15.4	Hemoglobin (mg/dL)	17.3	Age (yrs.)	12	BMI (kg/m^2^)	18
PaFi (mmHg)	11.1	% Neutrophils	15.2	Hematocrit (%)	17	BMI (kg/m^2^)	11.7	Weight (kg)	16.7
SaFi (mmHg)	10.7	Neutrophils (mcL)	10.4	Erythrocytes (mcL)	12.2	CRP (mg/dL)	10.2	CRP (mg/dL)	7.7
Altitude	9.7	Leukocytes (mcL)	7.4	Temperature (°C)	8	Weight (kg)	7.8	Leukocytes (mcL)	7.4
MCV (fL)	8.4	Lymphocytes (mcL)	6.4			RDW (%)	7.1	Neutrophils (mcL)	6.7
pO_2_ (mmHg)	7.1	% Monocytes	5.8			EPO (mUI/mL)	7	Lymphocytes (mcL)	5.6
SOFA	5.8	Hemoglobin (mg/dL)	5.6			% Basophils	6	APACHE II	5.2
		% Basophils	5.5						

PC6		PC7		PC8		PC9		PC10	
Eigenvalue = 1.8		Eigenvalue = 1.7		Eigenvalue = 1.4		Eigenvalue = 1.3		Eigenvalue = 1.1	
Variable	%	Variable	%	Variable	%	Variable	%	Variable	%
EPO (mUI/mL)	14.5	pCO_2_ (mmHg)	26.9	Heart rate (BPM)	22	Age (yrs.)	20.2	MCH (pg)	14.6
SaO_2_ (%)	9.1	Days in ICU	8.5	Days in ICU	17.9	Days in ICU	14.4	% Eosinophils	13.9
MAP (mmHg)	8.5	Temperature (°C)	7.8	MCH (pg)	6.7	APACHE II	9.5	Neutrophils (mcL)	8
Lymphocytes (mcL)	8.3	MAP (mmHg)	6.7	RDW (%)	6.3	SOFA	7.8	% Monocytes	7.7
Heart rate (BPM)	7.5	Leukocytes (mcL)	5.8	CRP (mg/dL)	5.2	Heart rate (BPM)	5.7	Weight (kg)	7.3
APACHE II	6.4	Lymphocytes (mcL)	5.6			% Eosinophils	5	APACHE II	6.1
% Basophils	6.1							Leukocytes (mcL)	5.8
Erythrocytes (mcL)	5.9							pCO_2_ (mmHg)	5.4
Temperature (°C)	5							Age (yrs.)	5.4

Only PCs, with eigen values greater than 1 are shown. Only variables with a contribution equal to or greater than 5% are shown.

**TABLE 6:** Clinical variables with predictive value for mortality in high-altitude patients with acute respiratory failure admitted to ICU.

	*p*-value	OR	95% CI	AUC	95% CI
Age (yrs.)	0.67	1	0.98–1.0	0.55	0.41–0.70
Weight (kg)	0.13	0.97	0.93–1.0	0.62	0.49–0.76
BMI (kg/m^2^)	0.48	0.98	0.89–1.1	0.61	0.47–0.75
Hb (mg/dL)	0.21	0.91	0.78–1.0	0.6	0.47–0.74
EPO (mUI/mL)	0.42	0.99	0.95–1.0	0.66	0.52–0.8
RDW (%)	0.26	0.94	0.84–1.0	0.56	0.42–0.70
Basophils (%)	**0.01**	0.15	0.031–0.5	0.72	0.60–0.84
Leukocytes (mcL)	**0.04**	1	1.0–1.0	0.65	0.52–0.79
Lymphocytes (mcL)	**0.05**	1	1.0–1.0	0.63	0.49–0.76
Lymphocytes (%)	**0.02**	0.92	0.85–0.98	0.69	0.57–0.82
Monocytes (%)	0.16	0.92	0.81–1.0	0.58	0.44–0.71
Neutrophils (mcL)	0.05	1	1.0–1.0	0.64	0.50–0.78
Neutrophils (%)	**0.01**	1.1	1.0–1.1	0.71	0.59–0.83
CRP (mg/dL)	**<0.0001**	1.1	1.1–1.2	0.82	0.71–0.92
APACHE II	**0.02**	1.1	1.0–1.2	0.67	0.54–0.81
SOFA	0.06	1.2	1.0–1.4	0.64	0.51–0.77

Significant p-values are shown in bold.

### The survival and mortality indicators identified in this study show no disparity among patients at both 2,640 and 4,150 m above sea level

Remarkably, our statistical analyses show no significant differences across the 48 clinical parameters examined between patients at 2,640 m (in Bogotá, Colombia) and 4,150 m (in El Alto, Bolivia) (Table 1). This finding should be interpreted with caution due to the limited availability of respiratory data, which precludes a definitive conclusion. Nonetheless, it may be suggested that living at altitudes above 2,600 m fosters a respiratory system sufficiently adapted to manage hypoxia, potentially reducing the reliance on ventilatory support for ARDS patients in the ICU compared to living at sea level. Additionally, it could be hypothesized that exposure to altitudes beyond 2,600 m does not significantly enhance the respiratory advantages already conferred by high-altitude living. However, further studies are essential to validate this hypothesis and to provide a more comprehensive understanding of the respiratory adaptations and limitations associated with extreme altitudes.

## Discussion

In this research, we performed a comparative analysis aimed at identifying clinical characteristics that impact the survival rates of intensive care unit (ICU) patients residing in two high-altitude regions: Bogotá, Colombia, located at an elevation of 2,640 m above sea level, and El Alto, Bolivia, located at an altitude of 4,150 m above sea level. The main findings of our study can be summarized as follows: first, C-reactive protein (CRP) emerges as the most reliable prognostic indicator for survival outcomes or mortality rates among ARDS ICU patients at high altitudes, with leukocytes, lymphocytes, basophils, neutrophils, and APACHE II score ranking second in relevance as significant indicators; second, respiratory and oxygenation parameters, such as PaO2/FiO2 or SaO2/FiO2 do not show an association with increased mortality in our study; third, no significant differences were found in this study depending on the altitude tested (2,640 and 4,150 m); fourth, no significant differences were found in this study depending on sex. Together, these results underscore the critical importance of revisiting existing guidelines for high-altitude ICUs, as they have been derived from settings that have not considered individuals who are adapted to permanent exposure to barometric hypoxia above 2,500 m.

Despite the compelling clinical significance uncovered by this study, which reveals the greater resilience of high-altitude permanent residents who are ICU patients with ARDS to hypoxic conditions, the findings are not surprising (Guo et al., 2023; Molano-Franco et al., 2023). Indeed, it is widely described in the scientific literature that chronic exposure to altitude hypoxia shapes intricate physiological, cellular, and molecular adaptations that allow gas exchange balance to be maintained (Horscroft et al., 2017; Mallet et al., 2023; Moore, 2017; Storz, 2021; Arias-Reyes et al., 2021b; Jochmans-Lemoine et al., 2018). In fact, it is these alterations that have facilitated successful human colonization of high-altitude regions (Zubieta-DeUrioste et al., 2023). However, these findings propel us into an area of opportunity, poised to revolutionize clinical care practices not only in high-altitude environments but also in sea-level hospitals. Indeed, the recalibration of guidelines designed specifically for high-altitude hospitals, with a special focus on improving inflammatory parameters, promises to substantially mitigate the persistently high mortality rates observed in ICU patients with ARDS. In parallel, investigating deeper into the intricate cellular and molecular mechanisms that underpin the greater resistance to hypoxia of high-altitude residents reveals an opportunity for possible new therapeutic strategies that may also be useful for sea-level ICU patients. In this context, erythropoietin deserves to be mentioned, a drug highly used in the clinic due to its safety and effectiveness (Ehrenreich et al., 2020; Jelkmann and Jelkmann, 2013). Indeed, erythropoietin, in addition to its erythropoietic property, is known to promote increased ventilation in hypoxic environments through direct interaction with brainstem centers that regulate ventilation (Soliz et al., 2005; Ballot et al., 2015; Soliz et al., 2009). Furthermore, we also know now that erythropoietin has anti-inflammatory, antioxidant, and angiogenic properties (Arcasoy, 2008; Nekoui and Blaise, 2017). In line, recent studies from our research group have shown a direct correlation between decreased serum erythropoietin levels and increased risk of mortality among high-altitude ICU patients with COVID-19 (Viruez-Soto et al., 2021). On the other hand, it is imperative to point out that scant attention is still being paid to unraveling the intricate mitochondrial adaptations generated by permanent residence at high altitudes. Adaptation to hypobaric hypoxia orchestrates the regulation of mechanisms that range from the modulation of oxidative stress, the regulation of inflammation, metabolic recalibration, and cellular differentiation to intercellular communication (Lee et al., 2020). Indeed, our previous investigations showed a striking contrast in mitochondrial efficiency between the liver and brain of mice (mammals adapted to high altitude) versus rats (mammals nonexistent above 2,500 m) (Arias-Reyes et al., 2021b; Arias-Reyes et al., 2023). Ultimately, a comprehensive investigation into the intricate cellular and molecular mechanisms, including the activation of hypoxia-inducible factors, enhancement of inflammatory responses, and reversal of mitochondrial dysfunction, which underpin the heightened resistance to hypoxia, is imperative (Rybnikova and Lukyanova, 2023).

A fascinating aspect of our research is the striking similarity of patient outcomes between altitudes of 2,650 m (Bogotá) and 4,150 m (El Alto). Surprisingly, despite the different altitudes, the physiological, cellular, and molecular adaptations induced by hypobaric hypoxia at these elevations appear to confer comparable benefits in terms of resistance to the complications of respiratory distress in ICU settings. Indeed, our data reveal that ICU patients with ARDS, regardless of altitude, exhibited elevated levels of C-reactive protein (CRP) and significant alterations in the levels of leukocytes, lymphocytes, basophils, and neutrophils among those who died from the condition. Based on these findings, it is tempting to speculate that individuals living at altitudes, whether healthy or sick, might be more susceptible to inflammation-induced injuries compared to those living at sea level. In fact, it has been suggested that acute mountain sickness, the etiology of which is still poorly understood, may be due to underlying inflammatory processes that are difficult to detect (Julian et al., 1985). In line with this, prescriptions of aspirin and ibuprofen have proven effective in preventing this condition (Burtscher et al., 1998; Xiong et al., 2017). Similarly, it has been suggested that chronic mountain sickness (a disease unique to high-altitude populations characterized by excessive erythrocytosis) may have its roots in inflammatory dysregulations that are challenging to identify (Villafuerte and Corante, 2016). Indeed, it is known that acetazolamide, used to prevent the progression of chronic mountain sickness, also has anti-inflammatory properties (Li et al., 2018; Shukralla et al., 2022).

This study had the inherent limitations of an observational cohort study, along with the conditions mentioned below. Although several respiratory parameters were assessed during the critical first 24 h of ICU admission, more detailed data on ventilatory mechanics are necessary to integrate both inflammatory and granular ventilatory variables. Such integration would provide a more comprehensive understanding of ARDS pathophysiology and its impact on patient outcomes. Additionally, the size of high-altitude hospitals presents challenges in recruiting large cohorts of patients. Despite this obstacle, our study successfully enrolled 52 patients in Bogotá and 18 patients in El Alto. Additionally, these pioneering efforts are expected to raise awareness among physicians and patients about the need to establish altitude-specific pathophysiological guidelines. We also anticipate that this work will promote greater participation in scientific studies from other hospital centers in the near future, improving the richness and integrity of our findings. It is also worth mentioning that the consistency of our study’s results in two hospital centers in different countries and altitudes supports the robustness of our conclusions.

Our study focused only on patients with ARDS and did not cover other potential etiologies. However, we anticipate that future research will delve deeper into these associated pathologies, increasing our understanding of the pathophysiology of high-altitude ICU patients.

Additional parameters, such as PEEP, lung mechanics, and duration of mechanical ventilation, were not thoroughly investigated in this study. Consequently, the mechanical ventilation strategy adopted in the two hospitals must account for altitude variances and the patient’s initial condition, including pulmonary hypertension and right ventricular function. This strategy should encompass low tidal volume, limitation of plateau pressure, and appropriate PEEP levels to minimize pulmonary stress, enhance PaO_2_ to counteract hypoxic pulmonary vasoconstriction, and mitigate hypercapnia. Hence, further exploration of these parameters is essential to discern their significance in high-altitude ICU settings and refine our approach to patient care within such environments.

In conclusion, the upcoming update of ARDS diagnostic and treatment criteria promises greater relevance and usefulness in the clinical setting. Indeed, this study shows that alterations in specific inflammatory markers are strongly associated with increased mortality, while oxygenation variables, including PaO2/FiO2 and SaO2/FiO2, do not show a significant correlation with mortality risk. These findings highlight the importance of focusing on inflammatory parameters when assessing prognosis in ARDS, particularly in high-altitude settings. This study is significant as it represents a pioneering initiative among high-altitude clinicians to identify critical clinical parameters that predict mortality or survival in patients with ARDS in high-altitude ICU settings. Looking ahead, with ongoing advancements in medical research, our findings underscore the necessity for more comprehensive studies to validate the direct impact of monitoring inflammatory parameters on patient survival. These findings will undoubtedly contribute to improving and refining existing medical protocols within high-altitude ICU facilities, addressing the unique challenges posed by the considerable resistance to hypoxia observed in the permanent inhabitants of these regions.

## Data Availability

The raw data supporting the conclusions of this article will be made available by the authors, without undue reservation.
